# Vegetation Mosaic Effects on Soil Microbial Community Structure and Enzyme Functioning in Relation to Nutrient Heterogeneity in a Mountainous Ecotone

**DOI:** 10.3390/plants15111672

**Published:** 2026-05-29

**Authors:** Gang Lei, Yang Yang, Wenting Li, Tian Chen, Lianghua Qi

**Affiliations:** 1 International Center for Bamboo and Rattan, Beijing 100102, China; leigang@icbr.ac.cn (G.L.); yangyang@icbr.ac.cn (Y.Y.); liwenting@icbr.ac.cn (W.L.); 2 Sanya Research Base, International Center for Bamboo and Rattan, Sanya 572000, China; 3 College of Life and Environmental Sciences, Huangshan University, Huangshan 245041, China

**Keywords:** vegetation mosaic, microbial community structure, soil enzyme activity, ecotone, nutrient heterogeneity

## Abstract

Vegetation mosaics characterize mountainous agroforestry ecosystems, yet how their spatial configuration shapes soil microbial assembly and functions remains unresolved. This study investigated how mosaic elements (monocultures, shrublands, and ecotones) drive microbial communities and enzyme activities across a forest–shrubland–farmland mosaic in western Hunan, China. Nutrient stoichiometry, microbial biomass (PLFA), and six enzyme activities were analyzed via variance partitioning, partial least squares regression, and ordination analysis. Fungal biomass dominated, peaking in ecotones and showing the lowest values in monocultures and shrublands. Microbial assembly was regulated by soil nutrients (31%) rather than soil texture (15%). Fungi (variable importance in projection, VIP = 1.287) and bacteria (VIP = 1.003) were key drivers, indicating distinct functional compartmentalization: fungi drove oxidative enzymes, whereas bacteria mediated nutrient cycling. Actinomycetes and total PLFA acted as secondary drivers, with VIP values of 0.932 and 0.939, respectively. Soil organic matter, dissolved organic carbon, silt content, and available nitrogen were key abiotic predictors. Collectively, vegetation configuration regulates soil functioning via nutrient-mediated microbial assembly and functional differentiation across mosaic elements. These findings underscore the role of landscape heterogeneity in sustaining soil fertility, suggesting that protecting ecotones and maintaining mosaic complexity should be prioritized in mountainous agroforestry management to enhance soil ecological functioning under global land-use change.

## 1. Introduction

Vegetation cover is a pivotal driver of soil physicochemical and biological processes [1], primarily mediated through litter quality, root exudates, and microenvironmental modification [2,3]. A consensus is emerging that the heterogeneity of substance inputs serves as the central mechanism by which vegetation configuration regulates soil nutrient cycling. For instance, mixed forestation has been shown to significantly enhance soil organic matter and sand particle proportions, an effect attributed to the optimized carbon-to-nitrogen ratio (C/N) of mixed litter and the increased transfer of decomposition products to the soil [4,5,6]. This underscores how vegetation mixing promotes carbon sequestration. The overarching influence of plant species composition is further highlighted by its ability to explain up to 96% of the variation in soil carbon fluxes [7]. Broadleaf litter, characterized by a lower C/N ratio than coniferous litter, contributes more effectively to soil carbon input and promotes a more uniform distribution of soil organic carbon (SOC) within the profile, thereby exerting a stronger positive effect on total nitrogen content in adjacent soils [1,8]. This indicates a coupled carbon–nitrogen regulatory mechanism in low C/N vegetation [9]. However, the regulatory role of vegetation remains highly context-dependent. Whereas Li et al. [6], Meier and Bowman [7], and Shankar and Garkoti [8] emphasize direct vegetation effects on soil biogeochemistry, He et al. [10] argue that regional environmental factors act as primary drivers, interacting with vegetation to shape soil biogeochemical heterogeneity. This discrepancy suggests that vegetation-driven nutrient cycling is not an isolated process but is modulated by broader environmental contexts, the effects of which are ultimately mediated by soil microbial metabolism. Thus, microbial community characteristics provide a critical lens through which to parse the heterogeneity of these regional drivers.

Soil microorganisms are central drivers of substance cycling and energy flow in terrestrial ecosystems [11,12]. Their community structure and metabolic functions collectively determine soil fertility and ecosystem functioning. Changes in vegetation type can significantly alter soil enzyme activities, largely through their effects on microbial community characteristics [13,14]. For example, shrubland often supports higher microbial biomass and phospholipid fatty acid (total PLFA), along with greater bacterial and fungal abundance compared to grasslands [15], due to improved microenvironments under shrubs, which enhance water retention and increase carbon and nitrogen inputs. In contrast, anthropogenic disturbances such as agricultural cultivation can reduce microbial biomass and diversity by destabilizing soil conditions and reducing organic matter inputs [16]. Vegetation types also shape the functional profile of soil enzymes by altering litter chemistry and soil microenvironments [17]. Urease activity, for instance, is generally higher in broadleaf forests, while amylase and sucrase activities are often greater in grasslands [18]. The conversion of broadleaf forests to *Cunninghamia lanceolata* (*C. lanceolata*) plantations has been shown to reduce sucrase, urease, and phosphatase activities but increase polyphenol oxidase activity [19]. Although soil microbial communities and enzyme activities are often tightly coupled, functional decoupling can occur under certain conditions. Talbot et al. [20] observed distinct fungal community structures across vegetation zones, despite similar extracellular enzyme activities, pointing to potential functional redundancy or differential microbial regulation. This raises a critical question: does microbial biomass reliably predict soil enzyme activities across heterogeneous vegetation configurations, or do specific vegetation types induce decoupling between community structure and functional expression?

Ecotones—transitional zones between vegetation types—are ideal systems for exploring soil–microbe–enzyme interactions due to their high environmental heterogeneity and strong edge effects. Understanding the ecological value of these transitional domains and their ripple effects is critical for revealing landscape-driven controls on soil biological processes [21,22]. Although previous studies have advanced our understanding of single vegetation types, how complex vegetation configurations in ecotones may continuously and nonlinearly affect belowground processes remains unclear, particularly regarding the relative contributions of chemical and physical drivers in shaping microbial communities. Furthermore, while nutrient availability and soil texture independently influence soil biota, their interactive effects on the community–function relationship in heterogeneous vegetation mosaics require further investigation. Although Huang et al. [23] linked leaf traits to soil organic carbon content, and He et al. [10] identified temperature as a common driver of nutrient cycling, neither study fully addressed how soil factors nonlinearly filter microbial communities and shape functional guilds in these transitional landscapes. Within these mosaics, the forest–shrubland–farmland interfaces experience complex gradients in resource availability and physical soil properties. Specifically, the continuous and nonlinear effects of vegetation configuration on belowground processes remain poorly understood. It remains unclear which dominates—chemical resources (nutrients) or physical habitats (texture)—in driving microbial functions, and how these factors interact to regulate community–enzyme relationships in transitional landscapes. This study focuses on a typical forest–shrubland–farmland mosaic in western Hunan, China. We systematically analyzed soil nutrients, microbial community structure, and enzyme activities across distinct vegetation zones and their ecotones. The objectives were to: (1) characterize spatial patterns of soil microbial communities and enzyme activities across the vegetation mosaic; (2) determine whether soil chemistry or soil texture predominates in driving microbial community assembly; and (3) examine the relationship between microbial community composition and soil enzymatic functioning. Elucidating these relationships is essential for predicting how landscape heterogeneity sustains soil fertility and for the management of ecotones and mosaic complexity in mountainous agroforestry systems under global land-use change.

## 2. Results

### 2.1. Soil Physicochemical Patterns Across the Vegetation Mosaic

**Figure 1:**
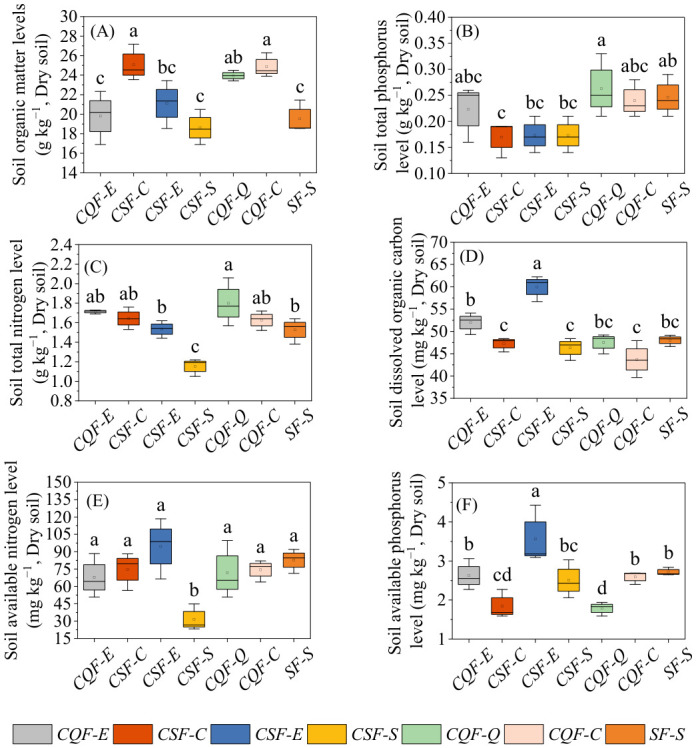
Spatial variation in soil nutrient availability across the forest–shrubland–farmland mosaic. Note: The labels (**A**–**F**) in the figure represent the spatial variability of soil organic matter, total P, total N, dissolved organic C, available N, and available P levels, respectively. The codes correspond to the following sampling sites: CQF-C: *Cunninghamia lanceolata* (*C. lanceolata*) of the *C. lanceolata*–*Q. fabri* (*Q. fabri*)–farmland zone; CQF-E: ecotone of the *C. lanceolata*–*Q. fabri*–farmland zone; CQF-Q: *Q. fabri* of the *C. lanceolata*–*Q. fabri*–farmland zone; CSF-C: *C. lanceolata* of the *C. lanceolata*–shrubland–farmland zone; CSF-E: ecotone of the *C. lanceolata*–shrubland–farmland zone; CSF-S: shrubland of the *C. lanceolata*–shrubland–farmland zone; SF-S: shrubland of the shrubland–farmland zone. Data in the figure are presented as mean ± SD. Different lowercase letters indicate significant differences at the 0.05 level as determined by one-way ANOVA.

As shown in Figure 1, vegetation type and sampling position significantly affected soil nutrient concentrations. SOM was significantly higher in *Q. fabri* forest than at the ecotone of CQF (*Cunninghamia lanceolata*–*Quercus fabri*–farmland gradient) and in shrubland (CSF: *Cunninghamia lanceolata*–shrubland–farmland gradient, SF: shrubland–farmland gradient; *p* < 0.05). SOM in *C. lanceolata* stands (CSF, CQF) was significantly higher than at the ecotone of CSF, whereas shrubland exhibited the lowest SOM. Dissolved organic carbon (DOC) was relatively elevated at the ecotones of CQF and CSF, but lowest in *C. lanceolata* stands and the shrubland plot within CSF. Total nitrogen (total N) was significantly higher in *Q. fabri* stands than at other sites (*p* < 0.05). The sites with shrub cover generally had lower total N, with the *C. lanceolata* of CSF recording the lowest values. Available N (AN) was significantly lower in the shrubland of CSF than elsewhere (*p* < 0.05). Total phosphorus (total P) was also lower in shrub-covered areas; shrubland in CSF had significantly lower total P than other sites (*p* < 0.05), while the *Q. fabri* of CQF had the highest total P, which was reduced under *C. lanceolata*. Consistent with DOC, available phosphorus (AP) was significantly higher at the ecotones in CQF and CSF than in the shrubland.

**Figure 2:**
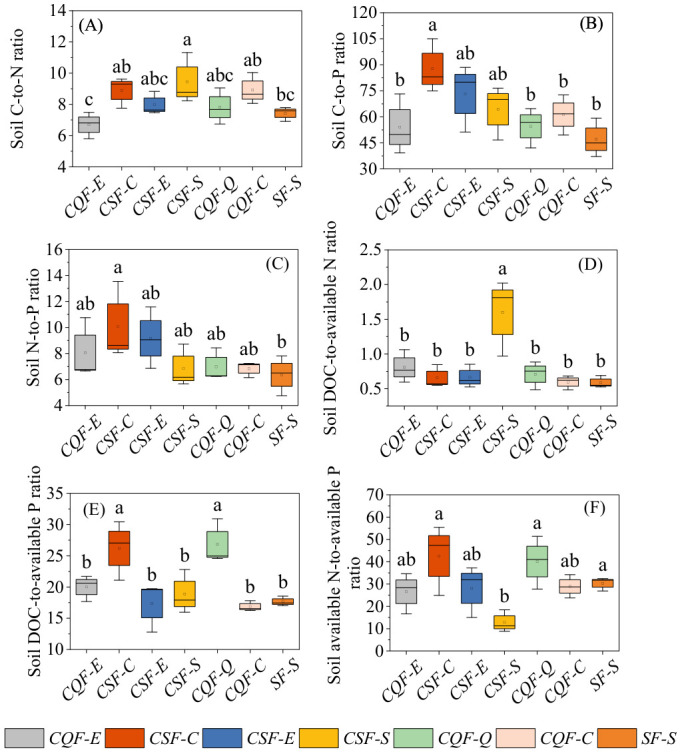
Spatial heterogeneity in soil ecological stoichiometry across the forest–shrubland–farmland mosaic. Note: The labels (**A**–**F**) in the figure represent the spatial variability of C-to-N ratio, C-to-P ratio, N-to-P ratio, soil dissolved organic C-to-available N ratio, soil dissolved organic C-to-available P ratio, and soil available N-to-available P ratio, respectively. The codes correspond to the following sampling sites: CQF-C: *Cunninghamia lanceolata* (*C. lanceolata*) of the *C. lanceolata*–*Q. fabri* (*Q. fabri*)–farmland zone; CQF-E: ecotone of the *C. lanceolata*–*Q. fabri*–farmland zone; CQF-Q: *Q. fabri* of the *C. lanceolata*–*Q. fabri*–farmland zone; CSF-C: *C. lanceolata* of the *C. lanceolata*–shrubland–farmland zone; CSF-E: ecotone of the *C. lanceolata*–shrubland–farmland zone; CSF-S: shrubland of the *C. lanceolata*–shrubland–farmland zone; SF-S: shrubland of the shrubland–farmland zone. Data in the figure are presented as mean ± SD. Different lowercase letters indicate significant differences at the 0.05 level as determined by one-way ANOVA.

As illustrated in Figure 2, vegetation type and sampling position strongly influenced soil ecological stoichiometry. The C/N ratio was highest in the shrubland of CSF, with significantly lower values in the shrubland of SF and CSF (*p* < 0.05). The C/P ratio peaked in the *C. lanceolata* of CSF, with the lowest values in the *Q. fabri* of CQF, the *C. lanceolata* and ecotone of CQF, as well as the shrubland of SF (*p* < 0.05). The N/P ratio was highest in *C. lanceolata* of CSF, with lower values at all other sites (*p* < 0.05). DOC/AN was distinctly higher in the shrubland of CSF than all other sites (*p* < 0.05). AN/AP was highest in the *C. lanceolata* of CSF, the *Q. fabri* of CQF and shrubland of SF, and lowest in the shrubland of CSF (*p* < 0.05).

**Figure 3:**
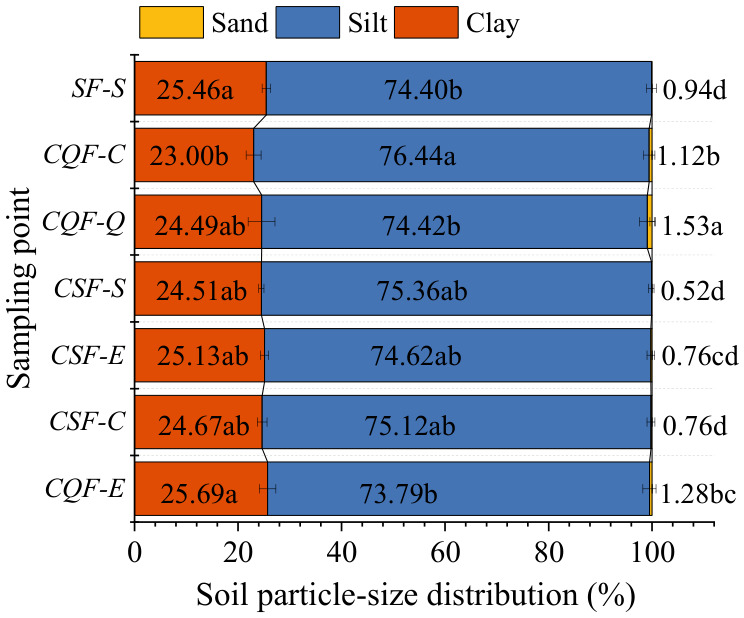
Soil particle-size composition across vegetation configuration zones. Note: The codes correspond to the following sampling sites: CQF-C: *Cunninghamia lanceolata* (*C. lanceolata*) of the *C. lanceolata*–*Q. fabri* (*Q. fabri*)–farmland zone; CQF-E: ecotone of the *C. lanceolata*–*Q. fabri*–farmland zone; CQF-Q: *Q. fabri* of the *C. lanceolata*–*Q. fabri*–farmland zone; CSF-C: *C. lanceolata* of the *C. lanceolata*–shrubland–farmland zone; CSF-E: ecotone of the *C. lanceolata*–shrubland–farmland zone; CSF-S: shrubland of the *C. lanceolata*–shrubland–farmland zone; SF-S: shrubland of the shrubland–farmland zone. Data in the figure are presented as mean ± SD. Different lowercase letters indicate significant differences at the 0.05 level, as determined by one-way ANOVA.

In addition, as seen in Figure 3, silt was the dominant particle-size fraction across all vegetation configuration zones, while clay content was consistently the lowest. The *C. lanceolata* of CQF had the lowest sand level among all sites (*p* < 0.05). Areas with *Q. fabri* growth exhibited higher clay content than other sites (*p* < 0.05).

### 2.2. Spatial Differentiation of Soil Microbial Communities

**Figure 4:**
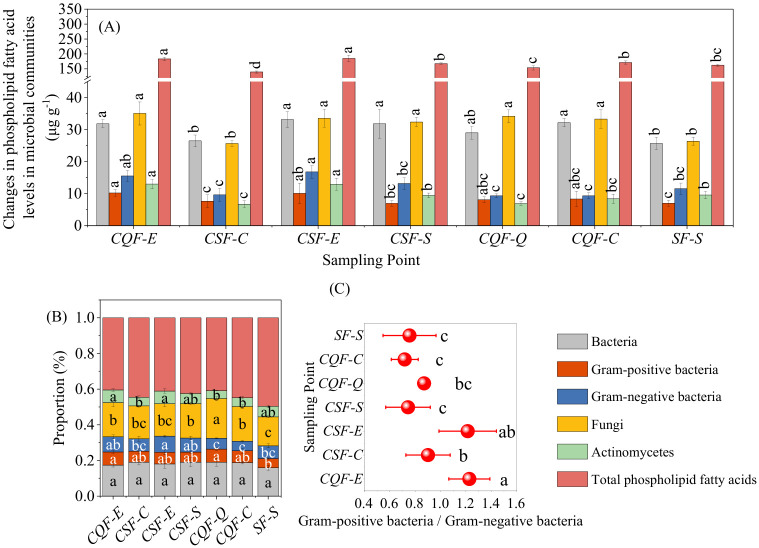
Spatial heterogeneity of soil microbial communities across the forest–shrubland–farmland mosaic. Note: Panels (**A**–**C**) represent the spatial heterogeneity of microbial phospholipid fatty acid (PLFA) levels, the changes in microbial community structure, and the ratio of Gram-positive to Gram-negative bacteria, respectively. The codes correspond to the following sampling sites: CQF-C: *Cunninghamia lanceolata* (*C. lanceolata*) of the *C. lanceolata*–*Q. fabri* (*Q. fabri*)–farmland zone; CQF-E: ecotone of the *C. lanceolata*–*Q. fabri*–farmland zone; CQF-Q: *Q. fabri* of the *C. lanceolata*–*Q. fabri*–farmland zone; CSF-C: C. *lanceolata* of the *C. lanceolata*–shrubland–farmland zone; CSF-E: ecotone of the *C. lanceolata*–shrubland–farmland zone; CSF-S: shrubland of the *C. lanceolata*–shrubland–farmland zone; SF-S: shrubland of the shrubland–farmland zone. Data in the figure are presented as mean ± SD. Different lowercase letters indicate significant differences at the 0.05 level as determined by one-way ANOVA.

A phospholipid fatty acid (PLFA) analysis (Figure 4) showed that vegetation type significantly affected soil microbial biomass and community structure. Total PLFA was higher in ecotones than in monocultures and shrublands, peaking in the ecotones of CQF and CSF, and reaching its lowest level in the *C. lanceolata* stand of CSF. Gram-positive bacterial (G^+^) PLFA was highest in the ecotones of CQF and CSF, as well as in the arbor forest of CQF, and lowest in the *C. lanceolata* of CSF and shrublands of SF. Gram-negative bacterial (G^−^) PLFA peaked in the ecotones in CQF and CSF and was lowest in the arbor forest of CSF. Total bacterial PLFA was highest in the ecotones of CQF and CSF, as well as the shrublands of CSF, and lowest in the *C. lanceolata* of CSF. The G^+^/G^−^ ratio was maximum in the ecotone of CQF and minimum in the shrublands of CSF and SF, as well as the *C. lanceolata* of CQF. Fungal PLFA was highest in the ecotones and lowest in the *C. lanceolata* of CSF and shrublands of SF. Fungi dominated in all vegetation zones, while actinomycetes accounted for the lowest proportion. Relative abundances of bacteria and actinomycetes were significantly higher in the ecotones of CQF and CSF. Fungal relative abundance was highest in the *Q. fabri* of CQF, where G^+^ and G^−^ relative abundances were the lowest.

**Figure 5:**
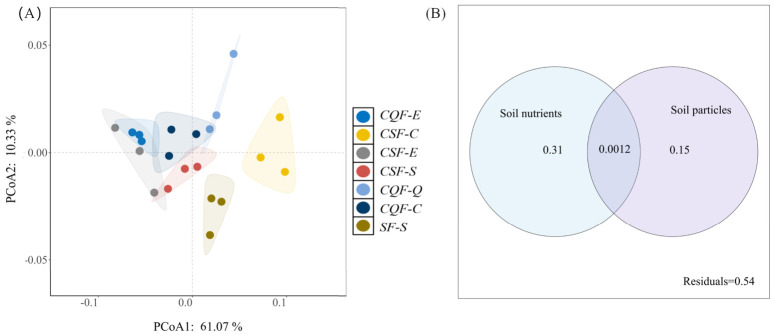
Principal coordinates analysis (PCoA) of soil microbial community structure (**A**) and variance partitioning analysis (VPA) for quantifying soil microbial variance based on soil nutrients and particle composition (**B**). Note: The codes correspond to the following sampling sites: CQF-C: *Cunninghamia lanceolata* (*C. lanceolata*) of the *C. lanceolata*–*Q. fabri* (*Q. fabri*)–farmland zone; CQF-E: ecotone of the *C. lanceolata*–*Q. fabri*–farmland zone; CQF-Q: *Q. fabri* of the *C. lanceolata*–*Q. fabri*–farmland zone; CSF-C: *C. lanceolata* of *C. lanceolata*–shrubland–farmland zone; CSF-E: ecotone of *C. lanceolata*–shrubland–farmland zone; CSF-S: shrubland of the *C. lanceolata*–shrubland–farmland zone; SF-S: shrubland of the shrubland–farmland zone. Data in the figure are presented as mean ± SD.

The principal coordinates analysis (PCoA) results of soil microbial PLFA profiles from the forest–shrubland–farmland composite ecosystem in western Hunan are shown in Figure 5A. The analysis revealed a clear separation of soil microbial community structures among the different vegetation configuration zones. PCoA1 and PCoA2 explained 61.07% and 10.33% of the total variation in soil microbial PLFA, respectively, with a cumulative explanatory rate of 71.40% for the first two axes. This indicates that the regulatory effect of vegetation configuration on the microbial community structure can be effectively characterized along these major coordinate dimensions. Figure 5B presents the results of the variance partitioning analysis (VPA). It identified soil nutrient elements and soil particle composition as factors influencing changes in microbial community structure, with significant differences in their contribution rates. Soil nutrient elements alone had an independent explanatory rate of 31% for the variation in microbial structure, suggesting that differences in SOM, N, P, and other nutrients are key drivers of the composition and structural differentiation of microbial communities. In contrast, soil particle composition had an independent explanatory rate of 15%. The joint explanatory rate of these two factors was merely 0.12%, indicating that soil nutrients and soil particles regulate the microbial community structure independently, without a significant interactive effect.

### 2.3. Variation in Soil Enzyme Activities

**Figure 6:**
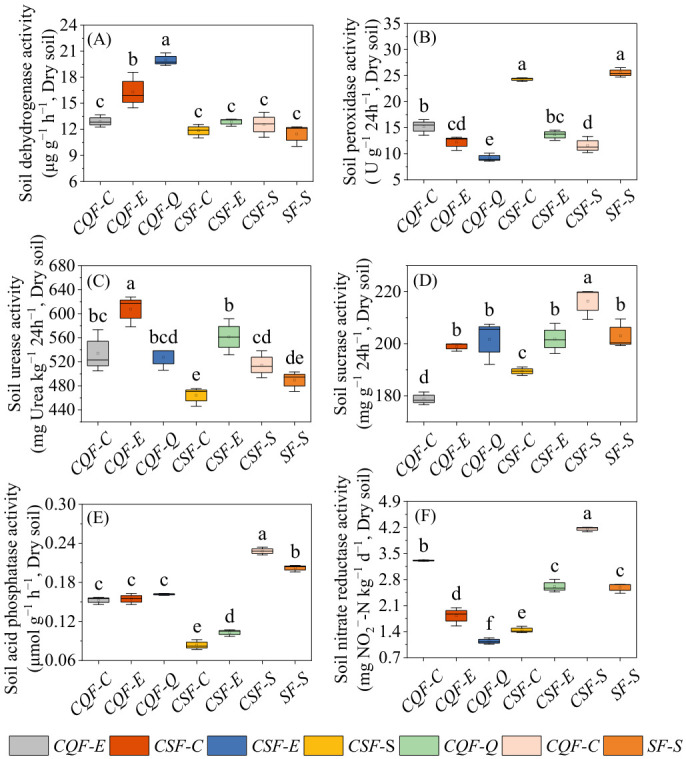
Spatial heterogeneity of soil enzyme activities across the forest–shrubland–farmland mosaic. Note: Panels (**A**–**F**) represent the spatial heterogeneity of soil enzyme activities, including dehydrogenase, peroxidase, urease, sucrase, acid phosphatase, and nitrate reductase. The codes correspond to the following sampling sites: CQF-C: *Cunninghamia lanceolata* (*C. lanceolata*) of the *C. lanceolata*–*Q. fabri* (*Q. fabri*)–farmland zone; CQF-E: ecotone of the *C. lanceolata*–*Q. fabri*–farmland zone; CQF-Q: *Q. fabri* of the *C. lanceolata*–*Q. fabri*–farmland zone; CSF-C: *C. lanceolata* of the *C. lanceolata*–shrubland–farmland zone; CSF-E: ecotone of the *C. lanceolata*–shrubland–farmland zone; CSF-S: shrubland of the *C. lanceolata*–shrubland–farmland zone; SF-S: shrubland of the shrubland–farmland zone. Data in the figure are presented as mean ± SD. Different lowercase letters indicate significant differences at the 0.05 level as determined by one-way ANOVA.

Analysis of six soil enzyme activities in the forest–shrubland–farmland ecotone of western Hunan (Figure 6) revealed highly specific responses of different enzyme types to vegetation configurations. Dehydrogenase and peroxidase exhibited contrasting spatial distribution patterns: dehydrogenase activity peaked in the *C. lanceolata* of CQF but dropped significantly to its lowest level at the ecotone of CQF. Conversely, peroxidase activity was minimal in the *Q. fabri* of CQF but reached its maximum in the *C. lanceolata* of CSF and shrubland of SF. Urease activity was highest at the ecotones of CQF and CSF, while it remained generally low in all shrub-dominated areas. Sucrase activity was significantly elevated in the shrubland areas but fell to its lowest level in the *C. lanceolata* of CQF. Both acid phosphatase and nitrate reductase activities peaked in the shrubland of CSF. Notably, acid phosphatase activity was lower in the *C. lanceolata*-dominated areas, whereas nitrate reductase activity showed a decline in the *Q. fabri*-grown regions.

**Figure 7:**
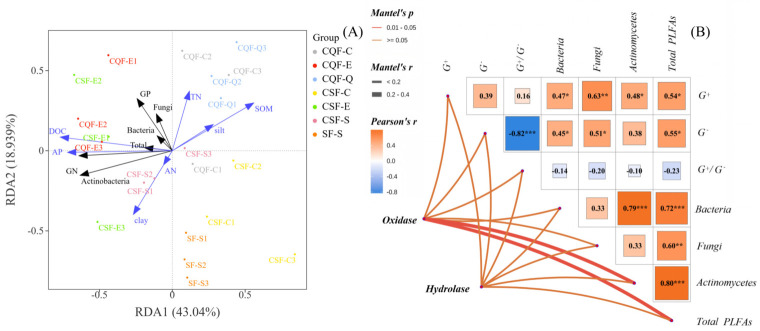
Redundancy analysis (RDA) of soil microbial community structure constrained by soil physicochemical properties (**A**) and Mantel–Pearson correlation analysis between soil enzyme activities and microbial community composition (**B**). Note: SOM: soil organic matter; TN: total nitrogen; AN: available nitrogen; AP: available phosphorus; DOC: dissolved organic carbon; silt: silt particle; clay: clay particle; total: total microorganisms; bacteria: bacterial PLFAs; fungi: fungal PLFAs; actinobacteria: actinobacterial PLFAs; GP/G^+^: Gram-positive bacteria; GN/G^−^: Gram-negative bacteria. The codes correspond to the following sampling sites: CQF-C: *C. lanceolata* of the *C. lanceolata*–*Q. fabri*–farmland zone; CQF-E: ecotone of the *C. lanceolata*–*Q. fabri*–farmland zone; CQF-Q: *Q. fabri* of the *C. lanceolata*–*Q. fabri*–farmland zone; CSF-C: *C. lanceolata* of the *C. lanceolata*–shrubland–farmland zone; CSF-E: ecotone of the *C. lanceolata*–shrubland–farmland zone; CSF-S: shrubland of the *C. lanceolata*–shrubland–farmland zone; SF-S: shrubland of the shrubland–farmland zone. The asterisks in panel (**B**) denote significance at the probability levels of *p* < 0.05 (*), *p* < 0.01 (**), and *p* < 0.001 (***).

A redundancy analysis (RDA) revealed distinct microbial community structures among the CQF, CSF, and SF-S sites (Figure 7A). The first two axes jointly explained 61.98% of the total variance, with RDA1 accounting for 43.04% and RDA2 for 18.94%. RDA1 correlated strongly with SOM, TN, and silt, reflecting soil nutrient availability, and was closely associated with CQF communities. RDA2 correlated with DOC, AP, and clay, indicating resource and texture heterogeneity, and separated ecotone areas, which had high DOC and AP, from SF-S/CSF-C, which exhibited high clay. AN contributed weakly to both axes. Microbial group vectors (bacteria, fungi, actinomycetes, total PLFAs) oriented toward the negative RDA1 direction, correlating positively with DOC and AP but negatively with SOM, TN, and silt. These axis-specific associations indicate that vegetation mosaic elements govern microbial assembly through nutrient-mediated niche partitioning. Pearson correlation analysis revealed a highly significant negative relationship between Gram-positive (G^+^) and Gram-negative (G^−^) bacteria (Figure 7B). G^+^ bacteria correlated positively with all microbial groups and total PLFAs, whereas G^−^ bacteria correlated positively with bacteria, fungi, and total PLFAs, but not with actinomycetes. Positive inter-group correlations identified actinomycetes as the primary contributor to total PLFA variation. The G^+^/G^−^ ratio showed no significant correlations with any indicator. These distinct correlation patterns provide a quantitative basis for understanding microbial biomass allocation strategies. These results indicate that the microbial community structure is tightly coupled with organic-matter hydrolysis processes, whereas its association with oxidative metabolism remains comparatively weak.

Partial least squares regression (PLSR) was used to evaluate the capacity of microbial indicators to predict soil enzyme activities. As summarized in Table 1, the cross-validation *R*^2^ values varied substantially among individual enzymes. Models for urease and peroxidase achieved relatively high explanatory power, with *R*^2^ values of 0.664 and 0.583, respectively, suggesting that microbial community structure could account for a major proportion of the variation in these two enzyme activities. In contrast, models for soil dehydrogenase, sucrase, acid phosphatase, and nitrate reductase exhibited low or even negative *R*^2^ values. This indicates that microbial properties alone have limited predictive capacity for these enzymes, which are likely governed more strongly by abiotic conditions. Variable importance in projection (VIP) scores further identified core microbial drivers. Table 2 shows that fungi (VIP = 1.287) and bacteria (VIP = 1.003) were identified as key variables, representing the primary biological regulators of soil enzyme function. Total PLFA, actinomycetes, G^−^, and G^+^ bacteria exhibited VIP values between 0.8 and 1.0, thus being classified as important but secondary variables. A canonical correlation analysis (CCA) further revealed the coupled structure between microbial communities and enzyme activities. Table 3 demonstrates that the first three canonical variables were statistically significant (*p* < 0.05), with the highest canonical correlation reaching 0.962 (*p* < 0.001). This indicates a strong, highly coordinated variation between microbial and enzyme variable sets, collectively suggesting that microbial regulation represents a key biogeochemical driver in the study area.

A stepwise multiple regression analysis was performed with soil nutrient properties and particle-size composition as independent variables and the activities of dehydrogenase, urease, sucrase, peroxidase, acid phosphatase, and nitrate reductase as dependent variables. The resulting regression equations are presented in Table 4. SOM was identified as the primary factor influencing dehydrogenase and urease activities. The regression model for dehydrogenase activity, with SOM as the sole predictor, yielded a coefficient of determination (*R*^2^) of 0.350 (*p* < 0.01). Similarly, SOM alone explained 27.5% of the variation in urease activity (*R*^2^ = 0.275, *p* < 0.05). Silt content and DOC were the key drivers of sucrase and peroxidase activities, respectively. Silt content accounted for 36.1% of the variation in sucrase activity (*R*^2^ = 0.361, *p* < 0.01), while DOC explained 40.0% of the variation in peroxidase activity (*R*^2^ = 0.400, *p* < 0.01). AN was a significant predictor of acid phosphatase activity, with a model *R*^2^ of 0.245 (*p* < 0.05). In contrast, nitrate reductase activity was jointly influenced by DOC and silt content; together, these two variables explained 82.1% of the variance in a highly significant regression model (*R*^2^ = 0.821, *p* < 0.01).

## 3. Discussion

### 3.1. Ecotones as Biogeochemical Reactors: Microbial Hotspots and Stoichiometric Subsidies

This study demonstrates that ecotones in forest–shrubland–farmland mosaics function as biogeochemical reactors where resource complementarity drives microbial biomass accumulation, but community restructuring optimizes functional efficiency [24,25]. Banerjee et al. reported increased extracellular enzyme activity across grassland–woodland ecotones driven by soil C and N availability [24], whereas Li et al. and Ren et al. collectively demonstrated that nutrient stoichiometry—whether manifested as microbial–substrate C:N:P imbalance or as exogenous N addition—governs decomposition rates and nitrogen conversion processes across forest–grassland and forest–steppe ecotones, respectively [25,26]. Our results extend this stoichiometric framework by demonstrating that natural nutrient subsidies in unmanaged subtropical ecotones activate comparable enzyme stimulation without exogenous inputs. Specifically, total PLFA, bacterial, and fungal biomass peaked at the ecotones of CQF and CSF (Figure 4A), accompanied by elevated DOC, AP, and urease activity (Figure 1E,F and Figure 6C). This confirms the edge effect theory [27,28] in subtropical montane ecosystems, extending its applicability beyond temperate grassland–forest transitions. However, unlike temperate systems where temperature and moisture gradients primarily govern edge effects, Qiu et al. reported that in lakeshore ecotones, moderate dry–wet alternation alleviates P limitation and enhances enzyme activities through hydrological pulsing [29]; by contrast, our montane ecotone operates as a static chemical gradient where lateral nutrient subsidies—not water-level dynamics—likely generate the stoichiometric subsidy effect, indicating that edge-effect mechanisms are context-dependent.

Mechanistically, the microbial advantage stems from asymmetric resource convergence: woody litter from *C. lanceolata* and *Q. fabri* forests provides structural carbon [30], while adjacent farmland inputs alleviate N and P limitation through fertilization legacies (Figure 1E,F). This creates a high-C, high-nutrient metabolic niche that enhances carbon use efficiency [31,32,33,34]. This compensatory mechanism is reflected in our mosaic, where the convergence of lignin-rich litter and fertilization-legacy nutrients creates a metabolic niche that sustains functional throughput. Notably, community restructuring at ecotones—evidenced by significantly higher relative abundances of bacteria and actinomycetes compared to monocultures (Figure 4B), despite fungal dominance across all zones—indicates trophic optimization, where fungal-driven carbon decomposition synergizes with bacterial nutrient mineralization. This fungal predominance in biomass (Figure 4B) aligns with their primary regulatory role in oxidative enzyme production (VIP = 1.287; Table 2), yet the moderate predictive power of fungal biomass alone (Table 1) suggests that specific functional expression still depends on community composition shifts and substrate stoichiometry [35]. Neurauter et al. showed that in subarctic birch–tundra ecotones, bacteria experience C, N, and P co-limitation, while fungi are constrained by C and N [36]; by contrast, our subtropical shrublands exhibit severe P depletion (Figure 2), where fungal oxidative efficiency appears constrained, and actinomycetes contribute to peroxidase residuals unexplained by fungal biomass alone. Li et al. further observed that in forest–grassland ecotones, microbes reallocate enzyme production from nutrient-acquisition to carbon-acquisition strategies in response to stoichiometric imbalance, and that simplified soil food webs emerge when C:N:P ratios decline [25]; analogously, the elevated C/N and C/P ratios in our shrubland patches correspond to reduced total PLFA and a compensatory shift toward actinomycete-mediated oxidation, suggesting that P limitation may trigger both enzyme reallocation and community restructuring. This explains the stoichiometric subsidy effect: despite higher microbial biomass, SOM accumulation was significantly lower in ecotones compared to adjacent *Q. fabri* forests (Figure 1A), reflecting efficient microbial carbon processing that enhances functional throughput at the expense of storage [37]. The contrasting trajectories of CQF and CSF gradients reveal context-dependent ecotone effects. In the CQF gradient (conifer–broadleaf–farmland), *Q. fabri* forests maintained the highest total P and N levels (Figure 1B,D), creating a nutrient legacy that subsidizes adjacent ecotones [38]. In contrast, the CSF gradient (conifer–shrubland–farmland) exhibited severe P depletion in shrubland (shrubland of CSF-S and SF-S) with distinct stoichiometric signatures (high C/N, low N/P; Figure 2). Fast-growing shrubs adopt a high N and P demand strategy [39], creating biological P sinks that exceed geochemical replenishment. Although acid phosphatase activity was elevated in shrubland (Figure 6E), mobilized P was rapidly sequestered in biomass, forming a mobilization–uptake–fixation loop that exacerbates soil P limitation. This pattern underscores the vulnerability of P-depleted shrublands in our mosaic.

Crucially, the PLSR analysis revealed context-dependent functional expression in degraded mosaic elements. While fungi were identified as key drivers of peroxidase activity (Table 2), their predictive power was moderate (Table 1), indicating that oxidative enzyme production is not solely governed by fungal biomass. The moderate predictive power of fungal biomass for peroxidase activity suggests that while fungi provide the structural capacity for lignin degradation, actual enzyme expression is modulated by additional factors—including community composition shifts (e.g., actinomycete abundance) and substrate stoichiometry (P limitation). This partial predictability creates niches for functional complementarity in resource-constrained microsites. Notably, in the phosphorus-limited shrubland of CSF and nutrient-depleted shrubland of SF, actinomycete abundance was positively associated with peroxidase residuals not explained by fungal biomass alone, suggesting localized functional contribution rather than universal differentiation. This pattern implies that P limitation may constrain fungal oxidative efficiency, yet actinomycetes maintain partial ligninolytic capacity through alternative metabolic pathways [40]. Consistent with the stoichiometric framework noted above, our observation that urease activity naturally peaks at ecotones suggests that unmanaged buffers can activate N-cycling enzyme–gene coupling through passive nutrient subsidy rather than active fertilization. For land management, these findings suggest that relying solely on fast-growing shrub monocultures in nutrient-depleted areas of western Hunan may compromise long-term soil fertility. This risk is especially pronounced under conditions of stoichiometric imbalance and reduced microbial biomass, which can degrade soil functions not only via phosphorus depletion (Figure 2) but also through functional imbalance within the decomposer community [41]. Mixed tree–shrub configurations that leverage the complementary functional traits—*Q. fabri*-derived fungal communities for lignin degradation (Table 2) and shrub-mediated P-mobilization via acid phosphatase (Figure 6E)—can optimize nutrient cycling without compromising ecosystem stability.

### 3.2. Hierarchical Filtering and the Architecture of Microbe Enzyme Coupling

Beyond spatial patterns at ecotones, vegetation configuration imposes hierarchical filters on belowground processes. Chen et al. demonstrated that in alpine treeline ecotones, microclimate—specifically freeze–thaw cycles and moisture availability—exerts stronger control over litter decomposition and lignocellulolytic enzyme activities than litter quality itself [42]; however, in our frost-free subtropical system, the absence of cryogenic disturbance decouples microbial assembly from physical stress, allowing nutrient stoichiometry to emerge as the dominant filter, whereas cryogenic or poorly evolved soils remain texture-limited. Variance partitioning analysis revealed that soil nutrients (31%) dominate microbial community assembly over texture (15%), with negligible interactive effects (0.12%; Figure 5B). This independence suggests that chemical resource availability and physical habitat operate as orthogonal niche axes—nutrients select for metabolic capacity, while texture determines spatial accessibility. Stepwise regression identified SOM as the foundational energetic driver for dehydrogenase (*R*^2^ = 0.350) and urease (*R*^2^ = 0.275), consistent with its dual role in energy provision and community regulation [18,43,44,45,46]. However, the negative coefficient for silt in the nitrate reductase model (*R*^2^ = 0.821) reveals a physical protection mechanism: fine silt particles enhance carbon occlusion, reducing bioavailability and suppressing carbon-dependent denitrification [44]. This generates a regulatory cascade: SOM determines functional potential for energy-intensive enzymes, while dissolved organic carbon (DOC, *R*^2^ = 0.400 for peroxidase) and particle-size composition (silt, *R*^2^ = 0.361 for sucrase) modulate specific oxidative and hydrolytic activities through substrate accessibility and physical protection (Table 4).

PLSR and CCA further resolved the architecture of coupling between microbiomes and enzyme functions. PLSR revealed a predictability threshold (cross-validation *R*^2^ > 0.5) separating two functional regimes: urease and peroxidase operate under biological control (*R*^2^ = 0.664 and 0.583, respectively; Table 1), whereas dehydrogenase, sucrase, acid phosphatase, and nitrate reductase fall below this threshold (negative or low *R*^2^), indicating environmental override where abiotic factors govern expression. Banerjee et al. reported that in forest–grassland ecotones, specific functional processes—including phosphorus cycling and nitrification—are directly associated with distinct microbial taxa, and that co-occurrence networks differ sharply between habitat patches [24]; our PLSR results corroborate this specificity by quantifying a predictability threshold that separates biologically controlled enzymes (urease, peroxidase) from environmentally overridden enzymes (dehydrogenase, sucrase, acid phosphatase, nitrate reductase). VIP scores identified fungi (VIP = 1.287) and bacteria (VIP = 1.003) as primary regulators under biological control (Table 2). However, the Mantel test qualifies this biological control by functional domain: hydrolase activities are significantly coupled with overall microbial community structure, whereas oxidase activities are not (Figure 7B). This pattern suggests that microbial community structure is tightly coupled with organic-matter hydrolysis, whereas its link to oxidative metabolism is comparatively weak; consequently, microbial composition likely modulates nutrient-acquisition hydrolysis, while oxidative carbon metabolism appears more responsive to direct abiotic substrate properties (Table 4). The convergence of VPA (nutrients explain 31% of community variation) and CCA (*p* < 0.001; Table 3) provides cross-scale validation: while chemical resources structure the microbiome (VPA), this structured community strongly coordinates with enzymatic functions (CCA). The mismatch between high canonical correlation (0.962) and moderate PLSR predictive power (maximum *R*^2^ = 0.664 for urease) further reveals that community composition alone is necessary but insufficient for functional prediction—specific functional guilds (actinomycetes) must be resolved to capture oxidative capacity. This coupling–differentiation continuum has profound implications for ecosystem prediction. Functional redundancy buffers carbon and nitrogen cycling against biodiversity loss (evidenced by bacteria–enzyme coupling), but lignin degradation involves contributions from specific functional guilds (actinomycetes, VIP = 0.93) in addition to fungal dominance [20]. In P-limited shrubland, this creates a functional vulnerability: despite high peroxidase activity maintained by actinomycete differentiation, persistent P depletion may eventually exhaust bacterial oxidative capacity, triggering carbon accumulation bottlenecks. Therefore, landscape configuration management must prioritize not only vegetation mixing but also stoichiometric balancing—maintaining adequate P availability to prevent the collapse of oxidative decomposition pathways in mosaic elements. Zheng et al. demonstrated that microbial community assembly in alpine treeline ecotones is regulated by litter quality and soil moisture at early decomposition stages, while pH and lignin content become dominant drivers in later stages [47]. Our single-timepoint sampling captures only the mid-summer metabolic profile shaped by nutrient availability, and we cannot rule out potential seasonal or decomposition-stage shifts in community structure mediated by pH and lignin dynamics. Beyond these temporal constraints, the substantial unexplained variance in microbial community assembly—beyond soil nutrients (31%) and texture (15%)—likely reflects additional drivers, including seasonal dynamics, plant–microbe interactions, and microscale spatial heterogeneity, which warrant future investigation through temporal sampling, root exudate characterization, and molecular functional profiling. Such approaches would further clarify the hierarchical controls on belowground biodiversity in mosaic landscapes, informing the management strategies outlined below.

Collectively, these findings establish vegetation mosaics as filtering landscapes in which nutrient heterogeneity structures microbial communities, and functional resilience emerges through community restructuring and functional differentiation rather than biomass accumulation alone. Building on this mechanistic understanding, we propose that strategic vegetation arrangement can serve as a generalizable tool for sustaining soil fertility across heterogeneous landscapes. Specifically, strategic vegetation management should configure mixed tree–shrub mosaics to exploit functional complementarity between fungal and bacterial guilds, conserve ecotones as biogeochemical buffers rather than converting them to pure stands, and employ stoichiometric monitoring to preemptively assess fertility decline risks. These practices collectively demonstrate that optimizing functional guild composition, rather than simply maximizing microbial biomass, can sustain soil ecological functioning across diverse mountainous agroforestry systems, offering a scalable framework for evidence-based management under global land-use change.

## 4. Materials and Methods

### 4.1. Study Area Overview

The study was conducted in Moshao Village (109°35′12″ E, 26°51′59″ N), Guangping Town, Huitong County, Huaihua City, Hunan Province, China. Located at the eastern edge of the Yunnan-Guizhou Plateau transition zone (430–470 m elevation), the site represents a typical subtropical mountainous ecotone. The study area features a 40–57° southeast-facing slope with distinct vertical vegetation zonation: *Cunninghamia lanceolata* plantations, native shrubland, and agricultural patches form a heterogeneous forest–shrubland–farmland mosaic (Figure S1). The region experiences a subtropical monsoon climate with a mean annual temperature of 16.8 °C (ranging from 4.8 °C in January to 26.7 °C in July). Approximately 70% of annual precipitation (1100–1400 mm) occurs during the concurrent heat-rain season (June–September). Soils derived from granite parent material are classified as Luvisols in the FAO soil classification system. These soils have a loamy sand texture and a pH of 4.4–5.3, typical of subtropical red soils [48].

### 4.2. Experimental Design and Sample Collection

The fieldwork was conducted in July 2024 within the designated study area. Within this vegetation mosaic, three gradients were established to represent different levels of compositional complexity: (1) a *Cunninghamia lanceolata*–*Quercus fabri*–farmland gradient (CQF); (2) a *Cunninghamia lanceolata*–shrubland–farmland gradient (CSF); and (3) a shrubland–farmland gradient (SF). A total of seven vegetation-type quadrats were deployed across these gradients. The CQF zone included quadrats of the *C. lanceolata* forest (CQF-C), ecotones (CQF-E), and *Q. fabri* forest (CQF-Q). The CSF zone comprised quadrats of the *C. lanceolata* forest (CSF-C), ecotones (CSF-E), and shrubland (CSF-S). The SF gradient comprised only a shrubland quadrat (SF-S), which provided key data on shrubland monoculture conditions for comparison with CSF-S (Figure S2). This experimental design captures mosaic-level heterogeneity, encompassing samples from monoculture stands (matrix patches) to ecotone interfaces. All quadrats were established with uniform dimensions of 3 m × 3 m, and each distinct vegetation type was replicated three times. A 5 m buffer zone was maintained between adjacent quadrats to avoid mutual interference. Furthermore, all quadrats were deliberately situated away from areas subject to farming, trampling, or other anthropogenic disturbances, with priority given to locations with homogeneous vegetation distribution and stable habitat conditions. Soil sampling was performed in August 2024. From each of the seven vegetation-type quadrats, soil samples were collected from the 0–20 cm depth layer using PVC tubes (5 cm in diameter, 20 cm in length). Four individual soil cores were extracted per quadrat and thoroughly mixed to form a single composite sample, which was representative of the quadrat. The composite samples were immediately placed in a cooling box for transport to the laboratory, where they were stored at 4 °C pending subsequent analysis. The collected soil samples were designated for the determination of a range of properties. These analyses encompassed soil physicochemical characteristics, including particle-size distribution and concentrations of carbon (C), nitrogen (N), and phosphorus (P). Additionally, the samples were used to assess biological indicators, namely soil microbial community structure and enzyme activities.

### 4.3. Sample Analysis Methods

#### 4.3.1. Analysis of Soil Physicochemical Properties

Prior to sampling, surface debris was removed. A subsample of each composite soil was passed through a 2 mm sieve, flash-frozen in liquid nitrogen, and stored at −20 °C for enzyme assays within one week. The remaining samples were transported to the laboratory, where they were air-dried, sieved (2 mm and 0.25 mm), and cleared of stones and root residues. Soil physicochemical properties, including soil organic matter (SOM), total nitrogen (TN), total phosphorus (TP), dissolved organic carbon (DOC), available nitrogen (AN), and available phosphorus (AP), were analyzed following *Soil Agricultural Chemistry Analysis* (3rd ed.) [49]. Measured soil enzyme activities included peroxidase [50], dehydrogenase [51], urease [52], sucrase [53], acid phosphatase [54], and nitrate reductase [55].

#### 4.3.2. Analysis of Soil Microbial Indicators

The methods described by Shen et al. [56], Zhang et al. [57], and Yang et al. [58] were adopted. Briefly, freeze-dried soil samples were homogenized and extracted twice with a citrate buffer solution, chloroform, and methanol mixture (volume ratio of 0.8:1:2) by vigorous shaking. The resulting extracts were subsequently separated using solid-phase extraction (SPE) silica gel columns to isolate fatty acids. The obtained phospholipid fatty acids (PLFA) were then subjected to alkaline methanolysis for derivatization into fatty acid methyl esters (FAMEs), with methyl nonadecanoate (19:0) employed as an internal standard. The FAME derivatives were analyzed using an Agilent 7890B gas chromatograph (GC; Agilent Technologies, Santa Clara, CA, USA) equipped with a flame ionization detector (FID). Quantification of individual PLFA was performed by referencing authentic fatty acid standards and the microbial identification system (MIDI, Inc., Newark, DE, USA). Microbial groups were differentiated based on biomarker PLFAs: General bacteria (represented by PLFA 14:0, 15:0, 15:0 DMA, 16:0, 17:0, 18:0, 20:0), Gram-positive (G^+^) bacteria (15:0 iso, 15:0 anteiso, 15:1 iso ω6c, 16:0 iso, 17:0 iso, 17:0 anteiso), Gram-negative (G^−^) bacteria (16:1 ω7c, 16:1 ω9c, 17:1 ω8c, 18:1 ω5c, 18:1 ω7c, 17:0 cyclo ω7c, 19:0 cyclo ω7c), fungi (18:1 ω9c, 18:2 ω6c), and actinomycetes (16:0 10-methyl, 17:0 10-methyl, 17:1 ω7c 10-methyl, 18:0 10-methyl, 18:1 ω7c 10-methyl).

### 4.4. Statistical Analysis

Data were analyzed using a one-way analysis of variance (ANOVA) and Duncan’s new multiple range test for multiple comparisons (*p* < 0.05) to examine differences among vegetation types. All results are expressed as mean ± standard deviation. Stepwise multiple regression analysis was employed to identify soil physicochemical factors influencing enzyme activities. These analyses were performed using SPSS 21.0 (SPSS Inc., Chicago, IL, USA) [59]. Variance partitioning analysis (VPA) was used to quantify the relative importance of soil nutrients versus particle composition in driving microbial community variation. Mantel tests were used to examine correlations between microbial community dissimilarity and soil property dissimilarity matrices. Principal coordinate analysis (PCoA) based on Bray–Curtis distance was conducted to visualize differences in soil microbial community structure across vegetation types. Partial least squares regression (PLSR) was used to evaluate the capacity of microbial indicators to predict soil enzyme activities. A canonical correlation analysis (CCA) was performed to reveal the coupled structure between microbial communities and enzyme activities. These multivariate analyses (VPA, Mantel tests, PCoA, PLSR, RDA, and CCA) were implemented in R 4.2.3 (R Foundation for Statistical Computing, Vienna, Austria) using the vegan, pls, and ggplot2 packages [60]. The figures were generated with Origin 2021 (OriginLab Corporation, Northampton, MA, USA).

## 5. Conclusions

Vegetation configuration structures belowground processes in forest–shrubland–farmland ecotones through nutrient-mediated microbial assembly. Ecotones accumulated DOC and AP, while shrubland areas reduced TN and TP, increasing C/N and C/P ratios. Microbial biomass PLFA was dominated by fungi across all vegetation zones, peaking at ecotones while declining in monocultures and shrublands, driven primarily by soil nutrients rather than texture. This fungal biomass dominance specifically underpinned oxidative enzyme activities, while bacteria and actinomycetes, despite their lower proportional abundance, functionally specialized in nutrient cycling, indicating functional differentiation aligned with community structure. Soil organic matter, dissolved organic carbon, and silt content emerged as hierarchical abiotic regulators. These findings support conserving ecotones as biogeochemical buffers rather than converting them to pure stands. Configuring mixed tree–shrub mosaics can exploit functional complementarity between fungal and bacterial guilds to sustain nutrient cycling, suggesting that management should prioritize stoichiometric balance and community composition over simply maximizing microbial biomass.

## Figures and Tables

**Table 1:** Predictive performance of the partial least squares regression (PLSR) model.

Enzyme Activity	Cross-Validation *R*^2^	MAE	RMSE	Usability
Dehydrogenase	0.145	0.757	0.925	Marginally usable
Peroxidase	0.583	0.547	0.646	Usable
Urease	0.664	0.479	0.580	Usable
Sucrase	−0.148	0.882	1.072	Unusable
Acid phosphatase	−0.357	0.921	1.165	Unusable
Nitrate reductase	−0.174	0.944	1.084	Unusable

Note: MAE = mean absolute error; RMSE = root mean square error. Usability was classified based on cross-validation *R*^2^: usable (*R*^2^ > 0.5), marginally usable (0 < *R*^2^ < 0.5), and unusable (*R*^2^ < 0).

**Table 2:** Variable importance projection (VIP) value ranking.

Rank	Microbial Indicator	VIP Value	Importance
1	Fungi	1.287	Key Variable
2	Bacteria	1.003	Key Variable
3	Total Phospholipid Fatty Acids	0.939	Important Variable
4	Actinomycetes	0.932	Important Variable
5	Gram-negative (G^−^)	0.915	Important Variable
6	Gram-positive (G^+^)	0.868	Important Variable

Note: VIP > 1 indicates key variables, and 0.8 < VIP < 1 indicates important variables.

**Table 3:** Canonical correlation coefficients and significance tests.

Canonical Variable Pair	Canonical Correlation Coefficient	Wilks’ λ	χ^2^	df	*p*-Value
Pair 1	0.962	0.0010	92.67	36	<0.001
Pair 2	0.947	0.0141	57.57	25	0.000
Pair 3	0.852	0.1354	26.99	16	0.042
Pair 4	0.666	0.4937	9.53	9	0.390
Pair 5	0.311	0.8881	1.60	4	0.809
Pair 6	0.130	0.9831	0.23	1	0.631

Note: Significance was set at *p* < 0.05. df = degrees of freedom.

**Table 4:** Stepwise regression analysis of soil enzyme activities based on soil nutrients and particle-size composition.

Soil Enzyme	Regression Equation	*p*	*R* ^2^	*F*
Dehydrogenase	0.879 + 0.600 × Soil Organic Matter	0.005	0.350	10.209
Peroxidase	−18.733 + 0.704 × Dissolved Organic Carbon	0.002	0.400	12.418
Urease	342.884 + 8.488 × Soil Organic Matter	0.015	0.275	7.220
Sucrase	650.552 − 6.036 × Silt	0.004	0.361	10.736
Acid phosphatase	0.084 + 0.001 × Available Nitrogen	0.023	0.245	6.163
Nitrate reductase	13.590 + 0.142 × Dissolved Organic Carbon − 0.242 × silt	0.001	0.821	41.339

## Data Availability

The original contributions presented in this study are included in the article/Supplementary Materials. Further inquiries can be directed to the corresponding authors.

## Supplementary Materials

The following supporting information can be downloaded at: https://www.mdpi.com/article/10.3390/plants15111672/s1, Figure S1: Sampling site overview; Figure S2: Sampling plot layout diagram.

## References

[1] Akbas M., Babur E., Tüfekçioglu A. (2026). Soil Physicochemical and Biochemical Differentiation Under Dominant Broadleaf Forest Species in the Eastern Black Sea Region. Forests.

[2] Lajtha K., Bowden R.D., Crow S., Fekete I., Kotroczó Z., Plante A.F., Simpson M.J., Nadelhoffer K.J. (2018). The detrital input and removal treatment (DIRT) network: Insights into soil carbon stabilization. Sci. Total Environ..

[3] Ma W.M., Tang S.H., Dengzeng Z., Zhang D., Zhang T., Ma X.L. (2022). Root exudates contribute to belowground ecosystem hotspots: A review. Front. Microbiol..

[4] Guo J.H., Kneeshaw D., Peng C.H., Wu Y.X., Feng L., Qu X.J., Wang W.F., Pan C., Feng H.L. (2025). Positive effects of species mixing on biodiversity of understory plant communities and soil health in forest plantations. Proc. Natl. Acad. Sci. USA.

[5] Guo X.Y., Yang G., Ma Y.X., Qiao S. (2024). Effects of different sand fixation plantations on soil properties in the Hunshandake Sandy Land, Eastern Inner Mongolia, China. Sci. Rep..

[6] Li S.T., Jiang Y.M., Lyu M., Deng C., Deng W., Wang M., Liu J.L., Lu Y.M., Xie J.S. (2024). High-quality litter exerts a greater effect on soil carbon gain in unrestored than restored pine plantations. Sci. Total Environ..

[7] Meier C.L., Bowman W.D. (2008). Links between plant litter chemistry, species diversity, and below-ground ecosystem function. Proc. Natl. Acad. Sci. USA.

[8] Shankar A., Garkoti S.C. (2023). Influence of forest types on soil physicochemical and biological characteristics of associated agroecosystems in the central Himalaya. Sci. Total Environ..

[9] Yao Y.W., Dai Q.H., Gao R.X., Yi X.S., Wang Y., Hu Z.Y. (2023). Characteristics and factors influencing soil organic carbon composition by vegetation type in spoil heaps. Front. Plant Sci..

[10] He Y.A., Bond-Lamberty B., Myers-Pigg A.N., Newcomer M.E., Ladau J., Holmquist J.R., Brown J.B., Falco N. (2024). Effects of spatial variability in vegetation phenology, climate, landcover, biodiversity, topography, and soil property on soil respiration across a coastal ecosystem. Heliyon.

[11] Chen J.C., Bai E., Liang Y.T., Liu Z.P., Ji Y.X., Sun T.T., Guo Z.X., Huo Y.D., Liu S.S., Berg B. (2025). The origin and succession of the microbial community in decomposing litter. ISME Commun..

[12] Yin P., Zhai K.Y., Zhao X.C., Li R.S., Fernández-Alonso M.J., Wang J., Yang H.X., Zhang X.Q., Berg B., Wang S.L. (2025). Microenvironmental variability differently predicts microorganism- and fauna-driven litter decomposition. J. Ecol..

[13] Bukombe B., Fiener P., Hoyt A.M., Kidinda L.K., Doetterl S. (2021). Heterotrophic soil respiration and carbon cycling in geochemically distinct African tropical forest soils. Soil.

[14] Qu H., Wang M.Y., Meng X.Y., Zhang Y.J., Gao X., Zhang Y.H., Sui X., Li M.H. (2025). Variations in the structure and composition of soil microbial communities of different forests in the Daxing’anling mountains, Northeastern China. Microorganisms.

[15] Fan Q.Y., Yang Y.G., Geng Y.Q., Wu Y.L., Niu Z.N. (2022). Biochemical composition and function of subalpine shrubland and meadow soil microbiomes in the Qilian Mountains, Qinghai-Tibetan plateau, China. Peer J..

[16] Camacho A., Mora C., Picazo A., Rochera C., Camacho-Santamans A., Morant D., Roca-Perez L., Ramos-Miras J.J., Rodriguez-Martin J.A., Boluda R. (2022). Effects of soil quality on the microbial community structure of poorly evolved mediterranean soils. Toxics.

[17] Yuan C.X., Wu F.Z., Wu Q.Q., Fornara D.A., Peng Y., Zhu G.Q., Zhao Z.M., Hedene P., Yue K. (2023). Vegetation restoration effects on soil carbon and nutrient concentrations and enzymatic activities in post-mining lands are mediated by mine type, climate, and former soil properties. Sci. Total Environ..

[18] Wang H.Y., Wu J.Q., Li G., Yan L.J. (2020). Changes in soil carbon fractions and enzyme activities under different vegetation types of the northern Loess Plateau. Ecol. Evol..

[19] Wang Y., Wang Y.M., Chen L.C. (2010). Effects of forest vegetation conversion on soil microbial biomass carbon and enzyme activity in Huitong, Hunan. Chin. J. Ecol..

[20] Talbot J.M., Bruns T.D., Taylor J.W., Smith D.P., Branco S., Glassman S.I., Erlandson S., Vilgalys R., Liao H.L., Smith M.E. (2014). Endemism and functional convergence across the North American soil mycobiome. Proc. Natl. Acad. Sci. USA.

[21] Babur E., Ozlu E., Uslu O.S. (2025). Soil respiration, microbial biomass, and stoichiometry within riparian buffers and adjacent land use. Sci. Rep..

[22] D’Odorico P., He Y.F., Collins S., De Wekker S.F.J., Engel V., Fuentes J.D. (2013). Vegetation-microclimate feedbacks in woodland-grassland ecotones. Glob. Ecol. Biogeogr..

[23] Huang X.M., Liu S.R., Wang H., Hu Z.D., Li Z.G., You Y.M. (2014). Changes of soil microbial biomass carbon and community composition through mixing nitrogen-fixing species with Eucalyptus urophylla in subtropical China. Soil Biol. Biochem..

[24] Banerjee S., Bora S., Thrall P.H., Richardson A.E. (2016). Soil C and N as causal factors of spatial variation in extracellular enzyme activity across grassland-woodland ecotones. Appl. Soil Ecol..

[25] Li B., Li Y.B., Li Q. (2023). Stoichiometric imbalances between soil microorganisms and their resources regulate litter decomposition. Funct. Ecol..

[26] Ren B.H., Li H.Y., Li D.Y., Meng M., Li J.H., Bai L., Feng Y.L. (2025). Nitrogen addition changes the nitrogen conversion process in forest steppe ecotone by increasing enzyme activity. Ecol. Process..

[27] Ma Y., Chen J., Li Z.Z., Zhou J.C., Zhang Y.X., Sun S.Y., Zhang W.X., Liu J.Z. (2025). The effect of soil physical structure in soil carbon and nitrogen distribution under different land use types in typical forest grassland transition zone on Loess Plateau, China. Soil Use Manag..

[28] Yin W.P., Guo X.M., Ma D.L., Yu H. (2025). Response of soil microbial communities between different vegetation types in the greater and lesser Khingan Mountains ecotone in northeast China. Microorganisms.

[29] Qiu M.S., Wang Y.W., Sun C.L., Gao X.Y. (2023). Dry-wet cycling area enhances soil ecosystem multifunctionality in the aquatic-terrestrial ecotones of the Caohai Lake in China. Environ. Sci. Pollut. Res..

[30] Shi L.Y., Zhang Y.T., Zhang L.J., Xu T.D., Zhao J.H., Li J.J., Yu C.Y., Guan Q.W. (2025). Arbor-shrub mixed vegetation restoration strategies enhanced soil organic carbon storage and stability via fine root and fungal characteristics in limestone hills. Plant Soil.

[31] Wang X.X., Zhang H.R., Cao D., Wu C.Y., Wang X.T., Wei L., Guo B., Wang S., Ding J.A., Chen H. (2024). Microbial carbon and phosphorus metabolism regulated by C:N:P stoichiometry stimulates organic carbon accumulation in agricultural soils. Soil Tillage Res..

[32] Wang C.Q., Kuzyakov Y. (2024). Soil organic matter priming: The pH effects. Glob. Change Biol..

[33] Zhao J.Y., Xie X., Jiang Y.Y., Li J.X., Fu Q., Qiu Y.B., Fu X.H., Yao Z.Y., Dai Z.M., Qiu Y.P. (2023). Effects of simulated warming on soil microbial community diversity and composition across diverse ecosystems. Sci. Total Environ..

[34] Li Y., Liang X.D., Yang N., Lin L., Gao T. (2025). Moisture-driven microbial regime shifts mediate nutrient dynamics in reservoir riparian zones. Water Res..

[35] Wang C.Q., Kuzyakov Y. (2024). Mechanisms and implications of bacterial-fungal competition for soil resources. ISME J..

[36] Neurauter M., Yuan M.Y., Rousk J. (2023). Soil microbial resource limitation along a subarctic ecotone from birch forest to tundra heath. Soil Biol. Biochem..

[37] Fu D.G., Wu X.N., Qiu Q.T., Duan C.Q., Jones D.L. (2020). Seasonal variations in soil microbial communities under different land restoration types in a subtropical mountains region, Southwest China. Appl. Soil Ecol..

[38] Li H.T., Li J.H., Mei L.L. (2025). Characteristics of Soil Nutrients and Microorganisms at the Grassland-Farmland Interface in the Songnen Agro-Pastoral Ecotone of Northeast China. Agronomy.

[39] Gao X.L., Li X.G., Zhao L., Kuzyakov Y. (2021). Shrubs magnify soil phosphorus depletion in Tibetan meadows: Conclusions from C:N:P stoichiometry and deep soil profiles. Sci. Total Environ..

[40] García-González I., García-Díaz A., Sastre B., Teutscherova N., Pérez M.A., Bienes R., Espejo R., Hontoria C. (2019). Mycorrhizal, nutritional and virgin olive oil parameters affected by groundcovers. J. Plant Nutr. Soil Sci..

[41] Xu G., Long Z.J., Ren P., Ren C.J., Cao Y., Huang Y., Hu S.L. (2020). Differential responses of soil hydrolytic and oxidative enzyme activities to the natural forest conversion. Sci. Total Environ..

[42] Chen Y.M., Liu Y., Zhang J., Yang W.Q., He R.L., Deng C.C. (2018). Microclimate exerts greater control over litter decomposition and enzyme activity than litter quality in an alpine forest-tundra ecotone. Sci. Rep..

[43] Waring B.G., Averill C., Hawkes C.V. (2013). Differences in fungal and bacterial physiology alter soil carbon and nitrogen cycling: Insights from meta-analysis and theoretical models. Ecol. Lett..

[44] de Menezes A.B., Prendergast-Miller M.T., Poonpatana P., Farrell M., Bissett A., Macdonald L.M., Toscas P., Richardson A.E., Thrall P.H. (2015). C/N ratio drives soil actinobacterial cellobiohydrolase gene diversity. Appl. Environ. Microbiol..

[45] Li J.Y., Ren T.B., Li Y.S., Chen N., Yin Q.Y., Li M.S., Liu H.B., Liu G.S. (2022). Organic materials with high C/N ratio: More beneficial to soil improvement and soil health. Biotechnol. Lett..

[46] Cai M.K., Cheng X.Q., Liu L., Peng X.H., Shang T.X., Han H.R. (2023). Soil microbial community and soil abiotic factors are linked to microorganisms’ C:N:P stoichiometry in larix plantations. Forests.

[47] Zheng H.F., Chen Y.M., Liu Y., Zhang J., Yang W.Q., Yang L., Li H.J., Wang L.F., Wu F.Z., Guo L. (2018). Litter quality drives the differentiation of microbial communities in the litter horizon across an alpine treeline ecotone in the eastern Tibetan Plateau. Sci. Rep..

[48] Lei G., Yang Y., Li W.T., Chen T., Qi L.H. (2026). Diameterclass-dependent species-specific tree–soil feedback linked to soil quality between *Cunninghamia lanceolata* (Lamb.) Hook. and *Quercus fabri* Hance in subtropical forests. Plants.

[49] Bao S.D. (2000). Soil and Agricultural Chemistry Analysis.

[50] Johnsen A.R., Jacobsen O.S. (2008). A quick and sensitive method for the quantification of peroxidase activity of organic surface soil from forests. Soil Biol. Biochem..

[51] Najdovski B., Djuric S., Pekec S., Milovic M., Orlovic S., Pilipovic A. (2025). Impact of systematic groups of microorganisms on dehydrogenase activity in soils within *Quercetum montanum* typicum forest community. Forests.

[52] Jiang D.Q., Wu C.R., Jiang N., Qiu W.W., Chen L.J., Zhang Y.L., Chen Z.H. (2025). Urease inhibitors increased soil intracellular urease activity while altering the ureC gene abundance and community structure in red soil. Appl. Soil Ecol..

[53] Deng J., Chong Y.J., Zhang D., Ren C.J., Zhao F.Z., Zhang X.X., Han X.H., Yang G.H. (2019). Temporal variations in soil enzyme activities and responses to land-use change in the Loess Plateau, China. Appl. Sci..

[54] Xiao S.H., You H.M., You W.B., Liu J.S., Cai C.T., Wu J.Q., Ji Z.R., Zhan S.H., Hu Z.S., Zhang Z.R. (2016). Rhizosphere and bulk soil enzyme activities in a Nothotsuga longibracteata forest in the Tianbaoyan National Nature Reserve, Fujian Province, China. J. For. Res..

[55] Li Z.J., Reichel R., Li Z.M., Yang K.J., Zhang L., Tan B., Yin R., Zhao K.R., Xu Z.F. (2022). Effects of snow absence on available N pools and enzyme activities within soil aggregates in a spruce forest on the eastern Tibetan Plateau. Soil Ecol. Lett..

[56] Shen H., Yan W.H., Yang X.Y., He X.H., Wang X., Zhang Y.T., Wang B., Xia Q.Y. (2020). Co-occurrence network analyses of rhizosphere soil microbial PLFA and metabolites over continuous cropping seasons in tobacco. Plant Soil.

[57] Zhang Y.Y., Zheng N.G., Wang J., Yao H.Y., Qiu Q.F., Chapman S.J. (2019). High turnover rate of free phospholipids in soil confirms the classic hypothesis of PLFA methodology. Soil Boil. Biochem..

[58] Yang Y.L., Xie H.T., Mao Z., Bao X.L., He H.B., Zhang X.D., Liang C. (2022). Fungi determine increased soil organic carbon more than bacteria through their necromass inputs in conservation tillage croplands. Soil Boil. Biochem..

[59] Kaya S., Erdogan Ç. (2007). The effect of operator’s characteristics over quality defects in sewing department in the apparel business. Tekst. Konfeksiyon.

[60] Lyu G., Hu J.Y., Ma J.C. (2024). Variation in Bacterial and Fungal Communities in Soils from Three Major Apple Pear (*Pyrus bretschneideri* Rehd.) Orchards. Microorganisms.

