# Supplementary figures and images for: Vegetation Mosaic Effects on Soil Microbial Community Structure and Enzyme Functioning in Relation to Nutrient Heterogeneity in a Mountainous Ecotone
Source: Plants (Basel). 2026 May 29;15(11):1672. doi: 10.3390/plants15111672 (PMC13258875; doi:10.3390/plants15111672)

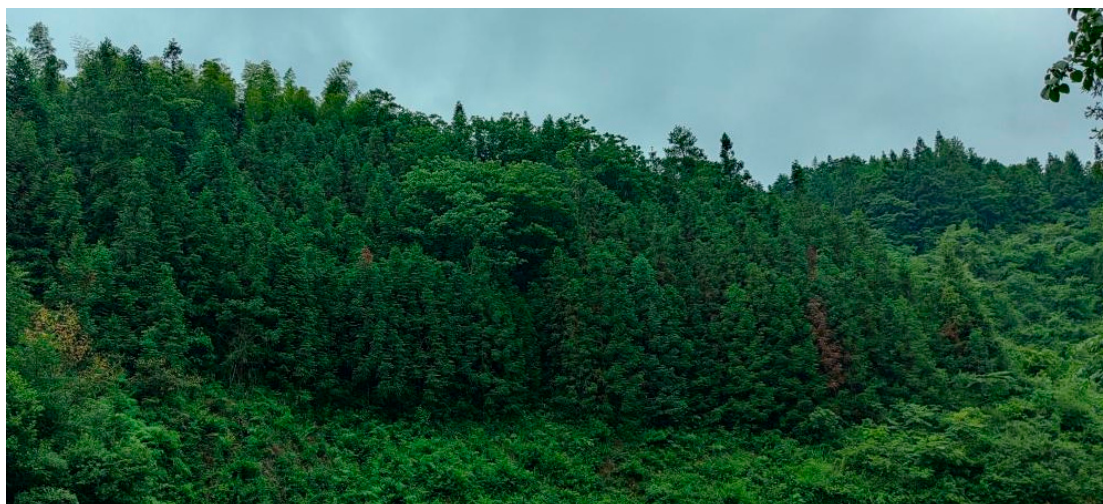

Figure S1 Sampling site overview

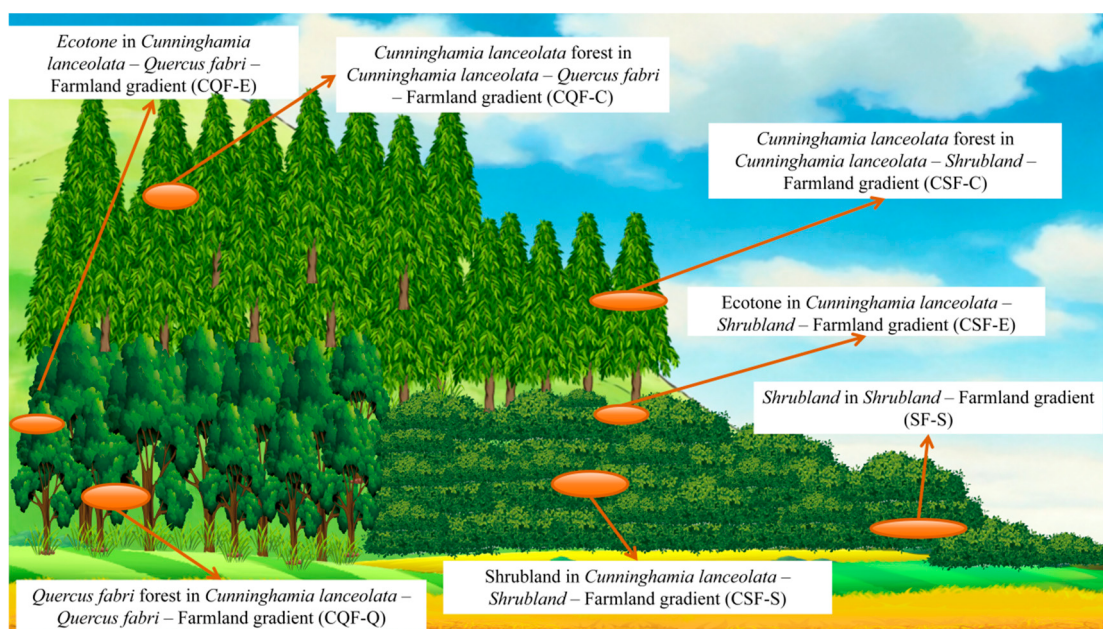

Figure S2 Sampling plot layout diagram

Supplement: Supplementary file 1 [file plants-15-01672-s001.zip › plants-4305660-supplementary.pdf]
